# Further Exploring the TRRAP Genotype–Phenotype Correlations: Report of Three New Patients With A Focus on Skeletal Anomalies

**DOI:** 10.1111/cge.70013

**Published:** 2025-06-29

**Authors:** Chiara Minotti, Sara Terreri, Andrea Del Fattore, Francesca Romana Lepri, Rosario Ruta, Maria Iascone, Laura Pezzoli, Maria Lisa Dentici, Antonio Novelli, Michelina Armando, Daniela Longo, Giuseppe Novelli, Domenico Barbuti, Andrea Bartuli, Ugo Cavallari, Ludovico Graziani, Maria Cristina Digilio, Lorenzo Sinibaldi

**Affiliations:** ^1^ Department of Biomedicine and Prevention Tor Vergata University Rome Italy; ^2^ Bone Physiopathology Research Unit, Translational Pediatrics and Clinical Genetics Research Division Bambino Gesù Children's Hospital, IRCCS Rome Italy; ^3^ Translational Cytogenomics Research Unit Bambino Gesù Children's Hospital, IRCCS Rome Italy; ^4^ Laboratorio di Genetica Medica ASST Papa Giovanni XXIII Bergamo Italy; ^5^ Rare Diseases and Medical Genetics Unit Bambino Gesù Children's Hospital, IRCSS Rome Italy; ^6^ UniCamillus Saint Camillus International University of Health Sciences Rome Italy; ^7^ Neuroscience and Neurorehabilitation Bambino Gesù Children's Hospital, IRCCS Rome Italy; ^8^ Neuroradiology Unit Bambino Gesù Children's Hospital, IRCCS Rome Italy; ^9^ Diagnostic and Interventional Radiology Bambino Gesù Children's Hospital, IRCCS Rome Italy; ^10^ Medical Genetics Unit, Laboratory Department Grande Ospedale Metropolitano Niguarda Milan Italy

**Keywords:** bone remodeling, neurodevelopmental delay, skeletal anomalies, *TRRAP*

## Abstract

*TRRAP* encodes a multidomain pseudokinase involved in histone acetyltransferase complexes. *TRRAP* pathogenic variants were linked to neurodevelopmental disorders, intellectual disability, congenital anomalies, and hearing loss. We report on three unrelated patients with *TRRAP* missense variants. Patient #1, a girl with severe intellectual disability, autism features, and preaxial polydactyly, displays the c.5575C>T, p.(Arg1859Cys) variant. Patient #2, a boy with developmental delay and facial anomalies, harbors the c.5647G>A, p.(Gly1883Arg) variant. Patient #3, a girl with developmental delay, epilepsy, and renal artery stenosis, carries the c.8572C>T, p.(Arg2858Trp) variant. These new cases broaden the *TRRAP* phenotypic spectrum, updating genotype–phenotype correlations. Osteoclast differentiation in Patient #1 and TRRAP expression in osteoclasts and osteoblasts were analyzed, leading to the assumption of a role of TRRAP in bone remodeling and in the observed skeletal anomalies.

## Graphical abstract

We report on the clinical characteristics of three new patients with pathogenic *TRRAP* variants expanding the syndrome's phenotype. In order to investigate the *TRRAP* potential involvement in skeletal development, osteoclastogenesis in Patient 1 was evaluated and TRRAP expression in osteoclasts and osteoblasts were analyzed.

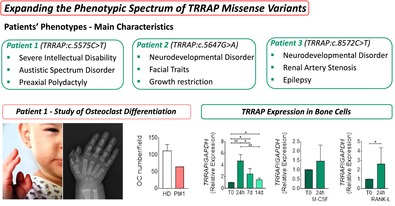

## 1 Introduction

*TRRAP* (MIM#603015) encodes a large, conserved multidomain pseudokinase, named TRansformation/tRanscription domain‐Associated Protein (TRRAP), in humans and other mammals, and Tra1 in yeast [1].

It mediates transcription activation by assembling histone acetyltransferase (HAT) complexes [2]. HATs specifically acetylate conserved lysine residues on histone tails, neutralizing their positive charges and loosening the nucleosome structures [2]. This biochemical variation is crucial for transcription, replication, and DNA repair [2].

*TRRAP* pathogenic missense variants were associated with developmental delay (DD), intellectual disability (ID), autistic spectrum disorder (ASD), and cranio‐facial features [3] (MIM#618454). Other characteristics, such as microcephaly and visual and hearing impairment, were observed in several unrelated patients [3].

*TRRAP* was also related to an autosomal dominant (AD) nonsyndromic hearing loss [4] (MIM#618778).

*TRRAP*'s function in development and genotype–phenotype correlations remains unclear due to the rare reports.

## 2 Clinical Reports

**FIGURE 1:**
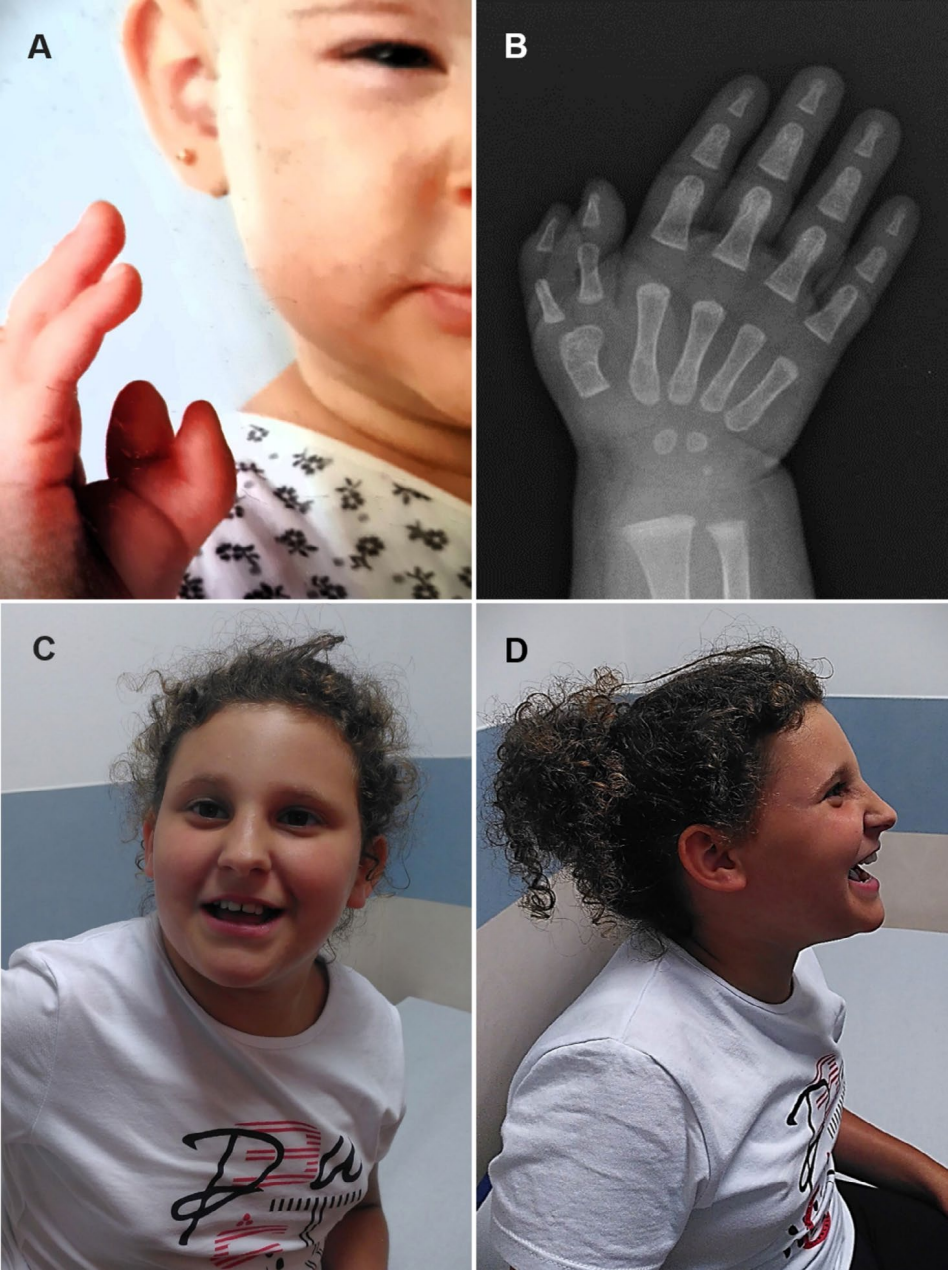
(A) Preaxial polydactyly (2 years). (B) X‐ray (1.5 years). (C) Horizontal eyebrows, hypotelorism, deeply set eyes, long nose, and short philtrum. (D) Prominent nasal bridge, long nose, and short philtrum. [Colour figure can be viewed at wileyonlinelibrary.com]

Patient #1 is a 10‐year‐old girl with DD, severe ID, ASD features, absent language, infancy onset epilepsy, and preaxial polydactyly of the right hand (Figure 1A,B). She showed peculiar facial characteristics (Figure 1C,D), tapered fingers, fifth finger bilateral clinodactyly, kyphoscoliosis, and bilateral pes planus. Brain magnetic resonance imaging (MRI) showed Chiari Malformation Type 1 (CM1). Exome sequencing (ES) revealed a *TRRAP de novo* pathogenic variant [NM_001244580.1: c.5575C>T; p.(Arg1859Cys)], according to American College of Medical Genetics and Genomics guidelines [5].

Patient #2 is a 5‐year‐old boy showing intrauterine growth restriction, developmental and growth delay, orofacial hypotonia, and severe astigmatism. At evaluation, he was able to spell a few words. His height and occipito‐frontal circumference were below −2 SDS. He displayed a triangular face, high anterior hairline, frontal bossing, small mouth, thin vermilion of upper and lower lips, protruding ears, and short, tapering fingers. ES demonstrated a *TRRAP de novo* missense likely pathogenic variant [NM_001244580.1: c.5647G>A; p.(Gly1883Arg)].

Patient #3 is an 11‐year‐old girl with DD, mild–moderate ID, epilepsy, and renal artery stenosis. Brain MRI showed a slight asymmetry of the lateral ventricles. Phenotypic evaluation showed a prominent forehead, mild hypertelorism, upslanting palpebral fissures, deeply set eyes, a prominent nasal bridge, a short philtrum, a thin upper lip vermilion, prominent finger pads, fourth to fifth toes bilateral clinodactyly, and generalized joint stiffness. ES identified a *TRRAP* novel *de novo* missense likely pathogenic variant [NM_001244580.1: c.8572C>T; p.(Arg2858Trp)].

## 3 Osteoclast Formation and TRRAP Expression in Bone Cells

**FIGURE 2:**
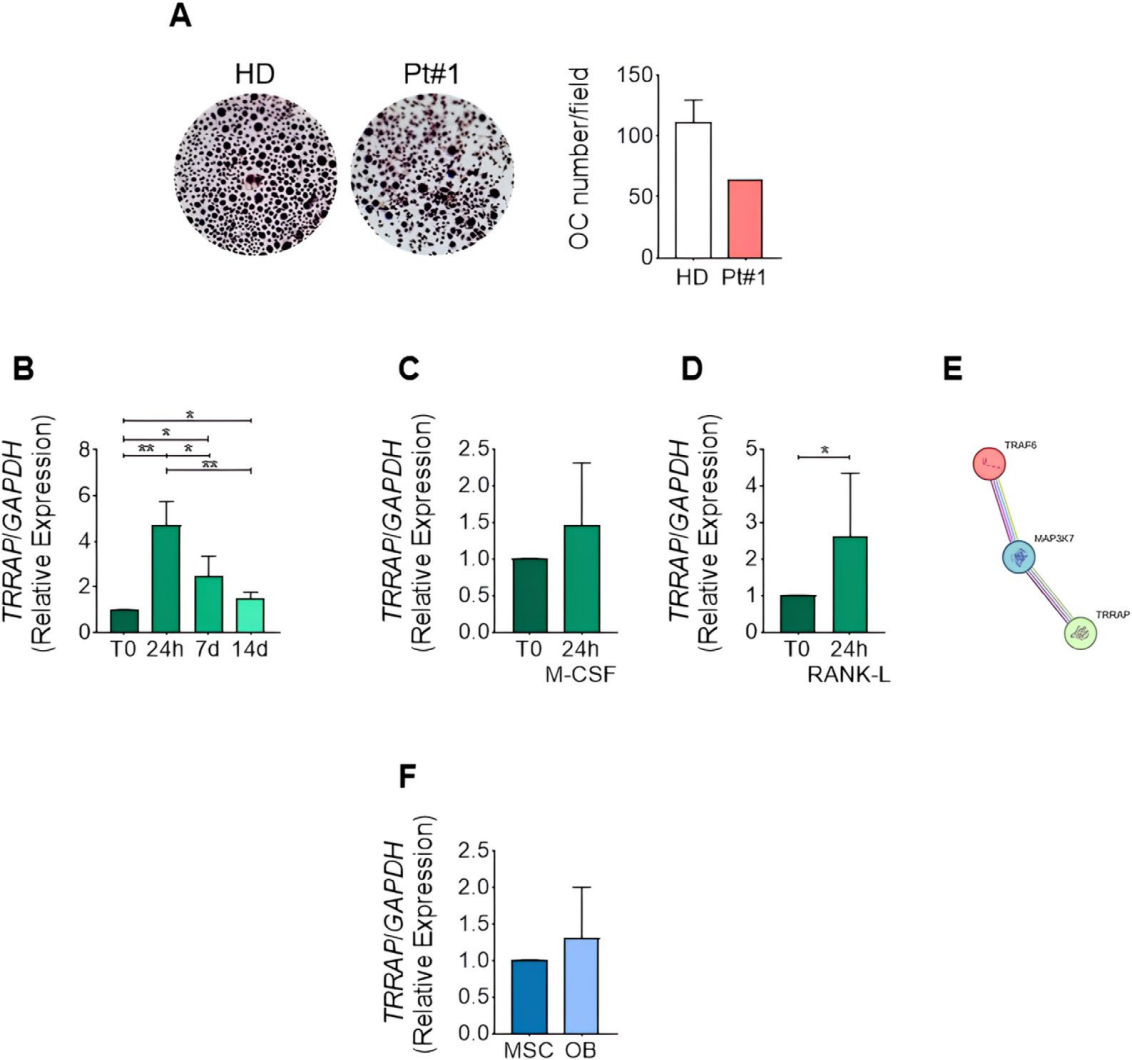
Osteoclast differentiation and TRRAP expression in bone cells. (A) Human adherent monocytes from Patient #1 and 3 healthy donors (HD) as controls were cultured with RANK‐L/M‐CSF for 14 days to differentiate into osteoclasts (OC). (Left panels) Tartrate‐resistant acid phosphatase (TRAcP) staining of osteoclast cultures. Original magnification ×10. (Right panel) Count of TRAcP positive OC/optical field. (B–D) Adherent monocytes of three HD were induced to differentiate into osteoclasts by M‐CSF and RANK‐L treatment. (B) RT‐PCR expression in monocytes (T0) and in cells treated with osteoclastogenic cytokines for 24 h, 7 days (osteoclast precursors) and 14 days (OC). (C, D) RT‐PCR expression analysis in monocytes treated for 24 h with (C) M‐CSF or (D) RANK‐L. (E) TRRAP‐MAP3K7 (TAK1)‐TRAF6 interaction in silico STRING analysis. (F) RT‐PCR expression in mesenchymal stem cells (MSC) and osteoblasts (OB). Results are expressed as mean ± SD. *p < 0.05, **p < 0.01. [Colour figure can be viewed at wileyonlinelibrary.com]

To investigate *TRRAP* involvement in skeletal development, we evaluated the effects of mutated TRRAP on osteoclastogenesis. Interestingly, mononuclear cells of Patient #1 showed ∼50% reduced ability to differentiate into osteoclasts compared to healthy donor cells (Figure 2A). To understand the role of TRRAP in osteoclast differentiation, we evaluated its levels in monocyte population before and after treatment with osteoclastogenic cytokines M‐CSF/RANK‐L (macrophage colony‐stimulating factor/receptor activator of nuclear factor‐κB ligand) for 24 h, 7 days (osteoclast precursors) and 14 days (multinucleated osteoclasts) (Figure 2B). RT‐PCR analysis revealed a peak of increase of TRRAP after 24 h stimulation with osteoclastogenic cytokines. Its levels reduce during osteoclastogenesis, but its expression in osteoclast precursors and osteoclasts is higher than in monocytes. Moreover, we observed that RANK‐L alone is sufficient to induce *TRRAP* expression during the early phase of osteoclastogenesis (Figure 2C,D). STRING analysis showed a tight association between TRAF6 (TNF receptor‐associated factor 6, involved in RANK‐L‐/RANK‐activated pathway) and TRRAP through the transforming growth factor‐β (TGF‐β)‐activated kinase (TAK1/MAP3K7) (Figure 2E). Gene expression analysis revealed that *TRRAP* is expressed also in osteoblasts and mesenchymal stem cells and is not modulated during osteoblastogenesis (Figure 2F).

## 4 Discussion

*TRRAP* pathogenic missense variants determine AD pleiotropic neurodevelopmental syndrome with or without peculiar facies and autism [3, 6, 7, 8] (MIM#618454).

*TRRAP* missense variants were associated also with neuropsychiatric disorders other than ASD, such as schizophrenia [9, 10] and childhood disintegrative disorder [11]. A de novo *TRRAP* missense variant was identified in a patient with very early onset psychosis, nonverbal learning disability, and history of obsessive‐compulsive disorder [12]. *TRRAP* missense variants were also associated with AD nonsyndromic hearing loss [4]. Zebrafish knockout/knockdown models showed significant inner ear defects and induced hair cell loss [4].

An intriguing genotype–phenotype correlation was uncovered. Cogné et al. outlined that patients with pathogenic variants involving amino‐acid residues 1031–1159 presented with a more severe, multisystemic disorder [3].

The three unrelated patients we report harbor different pathogenic variants located outside the 1031–1159 cluster. Noteworthy, two of the here reported variants were previously described, suggesting the possible existence of mutational hotspots. In Supporting Information and Table S1 clinical features of patients displaying the same pathogenic variant are discussed. Of note, Patient #1's clinical picture is severe compared to other patients with variants mapping outside the 1031–1159 cluster.

The multiple congenital malformations recently reported in two patients by Reyna‐Fabián et al. and Clay et al. [7, 8] are consistent with the hypothesis of a more severe phenotype in the 1031–1159 cluster. On the other hand, the case described by Acharya et al. showed multiple malformations and brain MRI anomalies [6], being the first syndromic case carrying a variant outside the 1031–1159 cluster.

A review of cases reported to date is presented in Table 1. Patients presented DD and 87% had a diagnosis of ID (100% inside the cluster 1031–1159 vs. 75% outside the cluster). Some clinical features have a similar prevalence between the two groups (i.e., variants inside and outside cluster 1031–1159), such as facial dysmorphisms (87% vs. 80%), short stature (38% vs. 28%), hypotonia (33% vs. 33%), and visual impairment (27% vs. 15%). Features predominantly found in the cluster 1031–1159 are microcephaly (53% vs. 7%), brain MRI anomalies (61% vs. 20%), cardiac malformations (64% vs. 33%), and genital malformations (47% vs. 7%). Cleft lip and palate, renal malformations, hearing impairment, and dysplastic nails were only described in patients with pathogenic variants in 1031–1159 residues. On the other hand, seizures have a prevalence of 40% outside the cluster (vs. 13%) and ASD was only associated with variants outside the cluster.

**TABLE 1:** Clinical phenotype of patients with pathogenic *TRRAP* variants.

	Cluster 1031–1159				Variants Outside the Cluster						All variants
Symptoms	Cogné et al. [3]	Clay [8]	Reyna‐Fabián et al. [7]	Total	Cogné et al. [3]	Acharya et al. [6]^a^	Patient #1, this report	Patient #2, this report	Patient #3, this report	Total	Total
Global DD	13/13	+	+	15/15 (100%)	11/11	+	+	+	+	15/15 (100%)	30/30 (100%)
ID	11/11	+	NA	12/12 (100%)	6/9	NA	+	+	+	9/12 (75%)	21/24 (87%)
Facial dysmorphisms	11/13	+	+	13/15 (87%)	8/11	+	+	+	+	12/15 (80%)	25/30 (83%)
ASD	0/13	−	NA	0/14 (0%)	5/11	NA	+	−	−	6/14 (43%)	6/28 (21%)
Microcephaly	6/13	+	+	8/15 (53%)	1/11	−	−	−	−	1/15 (7%)	9/30 (30%)
Short stature	4/12	+	NA	5/13 (38%)	3/11	NA	−	+	−	4/14 (28%)	9/27 (33%)
Hypotonia	4/13	−	+	5/15 (33%)	4/11	+	−	−	−	5/15 (33%)	10/30 (33%)
Feeding difficulties	7/13	+	−	8/15 (53%)	1/11	+	−	+	−	3/15 (20%)	11/30 (27%)
Seizures	1/13	−	+	2/15 (13%)	4/11	−	+	−	+	6/15 (40%)	8/30 (27%)
Cleft lip and palate	5/13	+	+	7/15 (47%)	0/11	−	−	−	−	0/15 (0%)	7/30 (23%)
Brain MRI anomalies	7/11	−	+	8/13 (61%)	0/7	+	+	NA	−	2/10 (20%)	10/23 (43%)
Cardiac malformations	9/12	−	−	9/14 (64%)	1/3	+	−	−	NA	2/6 (33%)	11/20 (55%)
Renal malformations	5/13	+	−	6/15 (40%)	0/4	−	−	NA	−	0/7 (0%)	6/22 (27%)
Genital malformations	5/13	+	+	7/15 (47%)	0/11	+	−	−	−	1/15 (7%)	8/30 (27%)
Hearing impairment	3/13	−	−	3/15 (20%)	0/11	−	−	−	−	0/15 (0%)	3/30 (10%)
Visual impairment	3/13	−	+	4/15 (27%)	1/11	NA	−	+	NA	2/13 (15%)	6/28 (21%)
Scoliosis	3/13	−	−	3/15 (20%)	0/11	NA	+	−	−	1/14 (7%)	4/29 (14%)
Dysplastic nails	8/13	−	−	8/15 (53%)	0/11	−	−	−	−	0/15 (0%)	8/30 (27%)
Lower‐limb hyperreflexia	1/13	−	−	1/15 (7%)	4/11	NA	−	−	−	4/14 (28%)	5/29 (17%)
Lacrimal‐duct aplasia	1/13	−	−	1/15 (7%)	2/11	−	−	−	−	2/15 (13%)	3/30 (10%)
Accessory nipple	3/13	−	−	3/15 (30%)	1/11	−	−	−	−	1/15 (7%)	4/30 (13%)

*Note:* Modified from Cogné et al. [3].

Abbreviations: ASD: autism spectrum disorder, DD: developmental delay, ID: intellectual disability, MRI: magnetic resonance imaging, NA: not applicable.

^a^ *TRRAP* (NM_001375524.1) c.11143G>A; p.(Asp3715Asn) [NM_001244580.1: c.11101G>A; p.(Asp3701Asn)].

We strengthen the effectiveness of a more severe and multisystemic disorder in patients with pathogenetic variants involving residues 1031–1159. Anyway, we highlight that patients harboring the same pathogenic variant show a significant variability, even outside the 1031–1159 cluster.

Lastly, to the best of our knowledge, we describe the first case of a patient with a pathogenic missense *TRRAP* variant and preaxial polydactyly. Noteworthy, patient n.21 by Cogné et al. presented with left hand postaxial polydactyly. Acharya et al. described a 3‐month‐old boy with nonspecified syndactyly and polydactyly [6].

Other unrelated patients described display minor skeletal anomalies, such as fifth finger clinodactyly, brachydactyly, second and third toe syndactyly, distal phalanges hypoplasia, small hands and feet, scoliosis/kyphoscoliosis, and vertebral anomalies [3]. Moreover, short stature in about 33% of the patients is a bone growth involvement feature of the disease (Table 1).

The skeletal features described in the here reported Patient #1 as well as the other described in precedent patients support a *TRRAP* role in skeletal development. As mentioned, *TRRAP* is indirectly involved in the acetylation process. Noteworthy, other neurodevelopmental disorders caused by abnormal histone acetylation show minor or major skeletal anomalies. Table S2 displays a clinical comparison of some of the most important syndromes associated with abnormal histone acetylation.

Previous studies in mouse models show that *Trrap* is expressed in forelimbs and hindlimbs [13, 14]. In a recent kinome‐wide CRISPR‐Cas9 library study, high expression of *TRRAP* in normal bone tissue and osteosarcoma cellular lines was demonstrated [15]. The present results show that *TRRAP* pathogenic variants affect osteoclast differentiation. In particular, the ability to differentiate into osteoclasts was ∼50% less in Patient #1's mononuclear cells when compared to healthy donor cells. We also observed that TRRAP is expressed by bone cells and it is induced by RANK‐L, which regulates osteoclast precursors' fusion into multinucleated osteoclasts. Since TRRAP reaches a peak of expression after RANK‐L 24 h stimulation, it could have a role in the initial commitment of monocytes into osteoclastogenic pathway. STRING analysis revealed a TRRAP potential involvement in RANK‐L induced osteoclastogenic pathway. RANK‐L binding RANK leads TRAF6 and TAK1 activation, conceivably associated with TRRAP. Interestingly, osteoclast‐specific TAK1‐knockout mice have an osteopetrotic phenotype, revealing that TAK1 is necessary for proper osteoclast differentiation [16]. Fronto‐metaphyseal Dysplasia is associated with *TAK1* de novo missense variants [17]. The present results lead to hypothesize a *TRRAP* role in bone remodeling, possibly associating to the reported skeletal anomalies. Further studies will dissect the *TRAAP* role in osteoclast differentiation and activity.

## 5 Conclusions

This study expands *TRRAP*'s phenotypic spectrum and suggests its role in skeletal anomalies. Clinical variability and nonspecific findings imply that *TRRAP*‐related disorders may be underdiagnosed. Thorough studies could explore *TRRAP's* genotype–phenotype correlations and role in osteoclastogenesis.

## Author Contributions

C.M., L.G., and L.S. elaborated the text. S.T. and A.D.F. analyzed osteoblast and osteoclast cultures. M.I., M.L.D., M.A., D.L., D.B., and U.C. overviewed the literature clinical reports. F.R.L., R.R., and L.P. overviewed the literature molecular data. A.N., G.N., and A.B. contributed to the overview of the text. M.C.D. and L.S. reviewed and edited the manuscript.

## Ethics Statement

This study was conducted according to the Declaration of Helsinki. It was reviewed and approved by the Institutional Review Board of IRCCS Bambino Gesù Children's Hospital (RRC‐2024‐3 875 028). Written informed consents were obtained from patients' parents for genetic testing and scientific publication.

## Consent

Written informed consent was obtained from the parents of the individual depicted in Figure 1.

## Conflicts of Interest

The authors declare no conflicts of interest.

## Supporting information


**Data S1.**Supporting Information.

**Table S1.** Clinical features of patients sharing the same pathogenic variant.

**Table S2.** Clinical comparison of syndromes associated with abnormal histone acetylation.

## Data Availability

The data that support the findings of this study are available on request from the corresponding author. The data are not publicly available due to privacy or ethical restrictions.
