# Supplementary material for: Further Exploring the TRRAP Genotype–Phenotype Correlations: Report of Three New Patients With A Focus on Skeletal Anomalies
Source: Clin Genet. 2025 Jun 29;109(1):181–7. doi: 10.1111/cge.70013 (PMC12674977; doi:10.1111/cge.70013)
Supplement: Supplementary file 1 — Data S1.Supporting Information. [file CGE-109-181-s001.docx]

**MATERIALS AND METHODS**

**Genetic analysis**

After genetic counseling, informed consent was obtained from patients’ parents for blood sampling. Karyotype analysis was performed on the metaphase chromosome preparations. CMA was performed using Infinium CytoSNP-850K BeadChip (Illumina, San Diego, CA), according to the manufacturer’s protocol. Array scanning data were generated on the Illumina NextSeq 550 system and the results were analyzed by the BluefuseMulti 4.4 software. Exome Sequencing was performed on genomic DNA using ClinEX pro kit (4bases, Manno, Switzerland) on the NovaSeq6000 platform (Illumina, San Diego, CA). In silico analysis was performed for coding regions and exon-intron junctions of genes associated with patients’ specific phenotypes.

**Osteoclast cultures**

Peripheral Blood Mononuclear Cells (PBMC) were isolated from EDTA-blood samples of patient#1 and healthy donors. Blood samples diluted in Phosphate Buffered Saline (PBS) solution was layered over Ficoll 1.077 g/ml (Lympholyte) centrifuged at 400 g for 30 min. “Buffy coat’’ was collected and washed twice with PBS. Cells were resuspended in DMEM medium containing 50 U/ml penicillin, 50 mg/ml streptomycin, 2 mM L-glutamine and 10 % FBS (Fetal Bovin Serum). Then 1 × 10^6^ cells/cm^2^ were plated on cell culture dishes or on bovine bone slices (IDS, PANTEC) to evaluate bone resorption activity. After 3 h, cell cultures were rinsed to remove non-adherent cells. Adherent monocytes were cultured in the presence of 20 ng/ml Macrophage Colony-Stimulating Factor (M-CSF, PeproTech, UK) and 30 ng/ml Receptor Activator of Nuclear factor kappa-B Ligand (RANKL, PeproTech, UK) for 14 days to form mature osteoclasts. To count osteoclasts, cells were fixed in paraformaldehyde and stained for TRAcP (Tartrate Resistant Acid Phosphatase) according to the manufacture’s instructions (kit #386, Sigma‐Aldrich, USA).

**Osteoblast cultures**

Bone Marrow Mesenchymal Stem Cells (MSC) were purchased from Lonza and were induced to differentiate in the presence of osteogenic medium (αMEM, 10% FBS, 50 U/ml penicillin, 50 mg/ml streptomycin and 2 mM L-glutamine supplemented with 10−7 M dexamethasone and 50 μg/ml L-ascorbic acid) for 21 days.

**Real time RT-PCR expression analysis**

Total RNA was extracted using the TriPure isolation reagent (Sigma-Aldrich, USA) and 1 μg was reverse transcribed and the equivalent of 50 ng was used for PCR reactions using SensiFAST SYBR Hi-ROX (Bioline, UK). Primers for the analysed gene and for the housekeeping gene GAPDH are the following: TRRAP Fw 5’-GTTGGAGCACCAGGCTTTTG-3’, TRRAP Rv 5’-GTGCTGCTCGTAAGCAATCG-3’; GAPDH Fw 5’-GGATTTGGTCGTATTGGG-3’, GAPDH Rv 5’-GGAAGATGGTGATGGGATT-3’. Each sample was analyzed in triplicate. The 2−ΔΔct method was used for relative quantitation of gene expression, and results are expressed as log10 (2−ΔΔct).

**Statistics**

Data were expressed as the mean ± s.d. of at least three independent experiments. Statistical analysis was performed by one-way analysis of variance, followed by the unpaired Student’s t-test or the Mann-Whitney U test. A p value < 0.05 was considered statistically significant.

**CASE REPORTS DESCRIPTIONS**

**Patient #1.** A 10-year-old girl was addressed to the Genetics Department due to neurodevelopmental delay, intellectual disability (ID) and absent language. She was the second child of healthy non-consanguineous parents with no remarkable family history.

She was born at 39 weeks by elective cesarean section, after an uneventful pregnancy. Birth weight was 2850 gr (-1.19 SDS), length was 48.0 cm (-0.99 SDS), and occipital frontal circumference (OFC) was 34.0 cm (-0.15 SDS). The Apgar score was 8-9. At birth, remarkable preaxial polydactyly of the right hand was evident.

X-ray of the right hand showed the presence of an accessory distal phalanx of the first finger. The preaxial polydactyly was surgically corrected at the age of six years.

The patient could sit without support at 12 months and walk independently at 16 months, with toe walking. At the time of the last examination, language was absent and she needed the support of augmentative/alternative communication.

At last examination (10 years and 10 months), the patient’s weight was 38 kg (-0.08 SDS), her height was 136 cm (-1.13 SDS) and OFC was 52 cm (-0.65 SDS).

Physical examination showed horizontal eyebrows, hypotelorism, deeply set eyes, prominent nasal bridge, long nose, short philtrum, bilateral clinodactyly of the 5th finger, tapered fingers, mild muscle hypertonia and poor motor coordination. She also presented kyphoscoliosis and bilateral pes planus with valgus hindfoot. Neuropsychiatric evaluation revealed severe ID, motor and vocal stereotypies, absent language, and autism spectrum disorder (ASD) features. Adaptive Behavior Assessment Scale II (ABAS-II) parents’ form was used at the age of 10 years to evaluate her adaptive skills. The global adaptive composite scores were 9 for the scaled score (SS) and 40 for the composite score (CS); conceptual adaptive domain scores were 3 for the SS and <57 for the CS; social adaptive domain scores were 2 for the SS and 50 for the CS; practical adaptive domain were 4 for the SS and 44 for the CS.

Her parents reported absence seizures when she was a child.  Electroencephalogram (EEG) showed epileptiform anomalies in the right fronto-centro-temporal hemisphere, involving the contralateral homologous regions. She is currently taking sodium valproate daily as a mood stabilizer.

She presented nocturnal and occasionally diurnal enuresis taking desmopressin at evening.

Ophthalmological evaluation, audiometric testing, abdominal ultrasound (US), echocardiography, spinal cord Magnetic Resonance Imaging (MRI) were normal, while brain MRI showed Chiari Malformation type 1 (CM1).

Standard karyotype and chromosomal microarray analysis (CMA) showed a paternally inherited 301 Kb deletion in 16q23.1 (GRCh37:78420775-78722387) encompassing *WWOX* gene, which is currently of uncertain significance. Clinical Exome Sequencing (CES), through Next Generation Sequencing (NGS), revealed a *TRRAP* *de novo* pathogenic variant (NM_001244580.1: c.5575C>T; p.Arg1859Cys), according to American College of Medical Genetics and Genomics (ACMG) guidelines^1^.

**Patient #2.** A 5-year-old boy was referred to the Genetics Department for developmental and constitutional growth delay. He was the second child of unrelated healthy parents.

He was born at 40 weeks via cesarean section. Intrauterine growth restriction was evident at the second-trimester fetal US examination. Birth weight was 2800 gr (-1.69 SDS). The Apgar score was 8-9. After birth, orofacial hypotonia was noted, with feeding difficulties and poor sucking.

He could walk at 18 months, and presented difficulties in complex mobility. Language development delay was reported. He was able to spell a few words at the time of examination.

At the last evaluation (5 years and 4 months) his height, weight, and OFC were 101.00 cm (-2.40 SDS), 14.60 kg (-2.55 SDS), and 51.5 cm (-0.94 SDS), respectively.

Physical examination disclosed triangular face, high anterior hairline, frontal bossing, small mouth, thin vermilion of upper and lower lips and protruding ears and short, tapering fingers.

Audiometric testing and echocardiography at four years of age were normal. Ophthalmological evaluation revealed severe astigmatism.

According to the Griffiths mental development scales (GMDS-III), the patient presented with a general quotient score of 79 and a development age of 34 months against a chronological age of 46 months, with the major deficits consisting of oculo-manual coordination, gross-motor, and language defects.

A normal 46, XY karyotype was documented. Methylation-specific MLPA (MS-MLPA) at 11p15, RP-PCR for CGG repetitions of the *FMR1* gene, and CMA analysis were negative. CES through NGS demonstrated a *TRRAP de novo* missense likely pathogenic variant (NM_001244580.1: c.5647G>A; p.Gly1883Arg), according to ACMG guidelines^1^.

**Patient #3** An 11-year-old girl was referred to the Genetics Department for neurodevelopmental delay, ID and generalized epilepsy. She was the first child of non-consanguineous healthy parents with unremarkable family history.

She was born at 38 week by elective caesarian section, after an uneventful pregnancy. Birth weight was 3050 g (-0.03 SD), length was 49 cm (0.15 SD), OFC was 33 cm (-0.49 SD). The Apgar score was 8-9.

Phenotypic evaluation showed prominent forehead, mild hypertelorism, upslanting palpebral fissures, deeply set eyes, prominent nasal bridge, short philtrum, thin vermilion of the upper lip, prominent finger pads, bilateral clinodactyly of IV and V toes and generalized joint stiffness.

Neurodevelopmental delay was noted and the neuropsychiatric evaluation revealed a mild-moderate ID.

According to WPPSI-III scale, the patient presented a total IQ of 74, with an uneven cognitive profile. Verbal cognitive abilities seemed less impaired, compared to nonverbal cognitive abilities, with a Verbal Intelligence Quotient of 86 and a Performance Intelligence Quotient of 70.

The patient also presented generalized epilepsy, with first episode at 2 years and 4 months. EEG showed generalized spike-wave discharges. She had been taking sodium valproate daily since diagnosis, but recently had to discontinue therapy due to hepatic steatosis on abdominal US, without subsequent seizures.

Brain MRI showed a slight asymmetry of the lateral ventricles, with the right side appearing larger.

At the age of 9 years a sudden increase in arterial blood pressure was noted. Color-Doppler ultrasonography showed a left renal artery stenosis. The stenosis was treated with percutaneous transluminal angioplasty, with a normalization of pressure values.

At the last examination (11 years and 10 months) her height, weight and OFC were 158 cm (1.05 SDS), 68.7 kg (1.94 SDS) and 56 cm (1.88 SDS), respectively. Pubertal development appeared normal: menarche had occurred at 11 years and 2 months.

CMA was negative. CES through NGS identified a novel *de novo* missense likely pathogenic variant in the TRRAP gene (NM_001244580.1: c.8572C>T; p.Arg2858Trp), according to ACMG guidelines^1^.

**CLINICAL FEATURES OF PATIENT HARBORING THE SAME PATHOGENIC VARIANT**

In Table S1 clinical features of patients sharing the same pathogenic variant are shown.

Patient #1’s phenotype [p.(Arg1859Cys)] is characterized by severe ID, absence seizures, ASD, absent language, peculiar facial characteristics, preaxial polydactyly, kyphoscoliosis and CM1. Patient n.17 reported by Cogné et al. harbors the same pathogenic variant with a much milder phenotype (ASD without associated anomalies)^2^. In contrast, patient #1’s clinical picture is severe when compared to other patients with variants mapping outside the 1031-1159 cluster.

Patient #2’s [p.(Gly1883Arg)] shows with DD, growth delay, peculiar facial features and feeding difficulties is consistent with the hypothesis of a less severe multisystemic involvement outside the 1031-1159 cluster. The same variant was described in patients n.20 and n. 21 by Cogné and colleagues^2^. We highlight the clinical variability among such patients. Patient n.20 only manifests DD and ASD, while patient n.21 presents DD, ASD traits, hypotonia, high reflexes in legs, peculiar facial characteristics, bilateral inguinal hernia, feeding difficulties, recurrent respiratory tract infections, strabismus, and postaxial polydactyly of the left hand.

**CLINICAL COMPARISON OF SOME OF SYNDROMES ASSOCIATED WITH ABNORMAL HISTONE ACETYLATION**

Other neurodevelopmental disorders caused by abnormal histone acetylation show minor or major skeletal anomalies. Table S2 displays a clinical comparison of some of the most important syndromes associated with abnormal histone acetylation. For example, in Rubinstein-Taybi syndrome (RSTS, MIM#180849 and 613684) a high prevalence of skeletal anomalies is noted, most often involving hands and feet, as broad and angulated thumbs and halluces are a major feature of this condition^5-7^. *KAT6B*-related disorders [Say-Barber-Biesecker-Young-Simpson syndrome (SBBYSS, MIM#603736) and Genito-Patellar syndrome (GPS, MIM#606170)] are associated with a wide range of skeletal anomalies, with absent/hypoplastic patellae, pelvic anomalies and a variety of hands and feet anomalies, including brachydactyly and preaxial and postaxial polydactyly (the latter was described only in SBBYS) ^8-12^. The neurodevelopmental disorder with central hypotonia and dysmorphic facies (NEDCHF, MIM#619797) associated with specific *HDAC4* missense variants was recently described, with 5/7 patients presenting spine anomalies (kyphosis/scoliosis)^4^. Cornelia de Lange syndrome, type 5 (CDLS5, MIM# 300882) is determined by loss of function *HDAC8* and it is associated with a variety of skeletal anomalies, mostly involving hands and feet^13^.

**REFERENCES**

[1]Richards S, Aziz N, Bale S et al. Standards and guidelines for the interpretation of sequence variants: a joint consensus recommendation of the American College of Medical Genetics and Genomics and the Association for Molecular Pathology. Genet Med. 2015 May;17(5):405-24. doi:10.1038/gim.2015.30.

[2]Cogné B, Ehresmann S, Beauregard-Lacroix E et al. Missense Variants in the Histone Acetyltransferase Complex Component Gene TRRAP Cause Autism and Syndromic Intellectual Disability. Am J Hum Genet. 2019 Mar 7;104(3):530-541. doi:10.1016/j.ajhg.2019.01.010.

[3]Haghshenas S, Bout HJ, Schijns JM et al. Menke-Hennekam syndrome; delineation of domain-specific subtypes with distinct clinical and DNA methylation profiles. HGG Adv. 2024 Jul 18;5(3):100287. doi:10.1016/j.xhgg.2024.100287.

[4]Wakeling E, McEntagart M, Bruccoleri M et al. Missense substitutions at a conserved 14-3-3 binding site in HDAC4 cause a novel intellectual disability syndrome. HGG Adv. 2021 Jan 14;2(1):100015. doi:10.1016/j.xhgg.2020.100015.

[5]Roelfsema JH, White SJ, Ariyürek Y et al. Genetic heterogeneity in Rubinstein-Taybi syndrome: mutations in both the CBP and EP300 genes cause disease. Am J Hum Genet. 2005 Apr;76(4):572-80. doi:10.1086/429130.

[6]Bartholdi D, Roelfsema JH, Papadia F et al. Genetic heterogeneity in Rubinstein-Taybi syndrome: delineation of the phenotype of the first patients carrying mutations in EP300. J Med Genet. 2007 May;44(5):327-33. doi:10.1136/jmg.2006.046698.

[7]Lacombe D, Bloch-Zupan A, Bredrup C et al. Diagnosis and management in Rubinstein-Taybi syndrome: first international consensus statement. J Med Genet. 2024 May 21;61(6):503-519. doi:10.1136/jmg-2023-109438.

[8]Clayton-Smith J, O'Sullivan J, Daly S et al. Whole-exome-sequencing identifies mutations in histone acetyltransferase gene KAT6B in individuals with the Say-Barber-Biesecker variant of Ohdo syndrome. Am J Hum Genet. 2011 Nov 11;89(5):675-81. doi:10.1016/j.ajhg.2011.10.008.

[9]Szakszon K, Salpietro C, Kakar N et al. De novo mutations of the gene encoding the histone acetyltransferase KAT6B in two patients with Say-Barber/Biesecker/Young-Simpson syndrome. Am J Med Genet A. 2013 Apr;161A(4):884-8. doi:10.1002/ajmg.a.35848.

[10]Campeau PM, Kim JC, Lu JT et al. Mutations in KAT6B, encoding a histone acetyltransferase, cause Genitopatellar syndrome. Am J Hum Genet. 2012 Feb 10;90(2):282-9. doi:10.1016/j.ajhg.2011.11.023.

[11]Simpson MA, Deshpande C, Dafou D et al. De novo mutations of the gene encoding the histone acetyltransferase KAT6B cause Genitopatellar syndrome. Am J Hum Genet. 2012 Feb 10;90(2):290-4. doi: 10.1016/j.ajhg.2011.11.024.

[12]Yabumoto M, Kianmahd J, Singh M et al. Novel variants in KAT6B spectrum of disorders expand our knowledge of clinical manifestations and molecular mechanisms. Mol Genet Genomic Med. 2021 Oct;9(10):e1809. doi:10.1002/mgg3.1809.

[13]Kaiser FJ, Ansari M, Braunholz D et al. Loss-of-function HDAC8 mutations cause a phenotypic spectrum of Cornelia de Lange syndrome-like features, ocular hypertelorism, large fontanelle and X-linked inheritance. Hum Mol Genet. 2014 Jun 1;23(11):2888-900. doi:10.1093/hmg/ddu002.

**TABLE LEGEND**

**Table S1**. Clinical features of patients sharing the same pathogenic variant.

**Table S2**. Clinical comparison of syndromes associated with abnormal histone acetylation.
